# Interplay between alpha and theta band activity enables management of perception-action representations for goal-directed behavior

**DOI:** 10.1038/s42003-023-04878-z

**Published:** 2023-05-06

**Authors:** Paul Wendiggensen, Astrid Prochnow, Charlotte Pscherer, Alexander Münchau, Christian Frings, Christian Beste

**Affiliations:** 1 Cognitive Neurophysiology, Department of Child and Adolescent Psychiatry, Faculty of Medicine of the TU Dresden, Dresden, Germany; 2 University Neuropsychology Center, Faculty of Medicine, Dresden, Germany; 3 Institute of Systems Motor Science, University of Lübeck, Lübeck, Germany; 4 Cognitive Psychology, Institute of Psychology, University of Trier, Trier, Germany

**Keywords:** Sensorimotor processing, Cognitive control, Human behaviour

## Abstract

Goal-directed behavior requires integrated mental representations of perceptions and actions. The neurophysiological underpinnings of these processes, however, are not yet understood. It is particularly undetermined, which oscillatory activities in which brain regions are involved in the management of perception-action representations. We examine this question with a focus on response inhibition processes and show that the dynamics of perception-action representations reflected in theta band activity (TBA) are particularly evident in the supplementary motor area and the occipito-temporal cortex. Mental representations coded in alpha band activity (ABA) during perception-action integration are associated with the occipito-temporal cortex. Crucially, perception-action representations are exchanged between theta and alpha frequency bands. The results imply that ABA functions as dynamic top-down control over binding, retrieval and reconfiguration processes during response inhibition, which in turn are reflected by TBA. Our study thus highlights how the interplay of oscillatory activity enables the management of perception-action representations for goal-directed behavior.

## Abstract (web summary)

Electrophysiological recordings in 79 human participants during a goal-directed task, reveals that an interplay between alpha and theta band activity enables management of perception-action representations.

## Introduction

The integration of perceptions and actions is central to goal-directed behavior. This is especially the case when ambiguous sensory information complicates response selection processes. Nevertheless, the question of how changes in the mental representations of stimulus-response associations are processed in cortical structures and which neurophysiological dynamics underlie these aspects remains elusive.

This conceptual question is important because influential cognitive science frameworks on the integration of perception and action focus on the *representation* of stimulus-response associations. The Theory of Event Coding (TEC), for example, provides a theoretical framework^1,2^, which delineates how perceptions become integrated (bound) with actions on a cognitive level and states that so-called event files (i.e., integrated representations of stimuli and their associated responses) are the mechanistic element behind goal-directed actions^2–4^. This binding of stimulus and action features facilitates performance if the same action is required in response to the (partially) same stimulus. However, on the contrary, if a different action is required in response to a similar stimulus, the features bound in the previously established event file need to be rebound to enable performance. This reconfiguration process is time-consuming and error-prone, leading to performance decreases which are called partial repetition costs^2,5^. Furthermore, the Binding and Retrieval of Action Coding (BRAC) framework^4^ emphasizes the dynamic handling of integrated perception-action representations (i.e., event files). Building on these theoretical frameworks, the neurophysiological and functional neuroanatomical basis of perception-action integration during response selection^6–10^ and inhibition^11–15^ have recently become increasingly well understood. Previous studies on event file coding processes in inhibition were able to validate the predictions regarding behavioral performance of TEC in this cognitive domain, i.e., the worse performance of Go and Nogo stimuli shared features than if they were completely distinct^11–15^. Further, these previous findings demonstrated the relevance of the theta and alpha frequency bands to event file coding in inhibition on the one hand by showing a seesaw-like relationship between both frequency bands^12,13^, and underlined the assumption that an event file can be understood as a mental representation of specific stimulus and response features^11,15^. Yet, the role of oscillatory brain activity in the management of event file representations remains elusive. It is therefore unknown how essential mechanisms regulating brain function (i.e., oscillatory activity)^16^ enable representational dynamics driving goal-directed actions.

It is assumed that representations integrating stimulus and response require distributed processing of stimulus and response features in network-like structures^2,3,9,12^. Thus, multiple cortical regions, concomitantly involved in goal-directed behavior, should show similar dynamics of stimulus-response representations. Even though this central conceptual aspect is evident for a long time, it has not been examined directly.

**Fig. 1:**
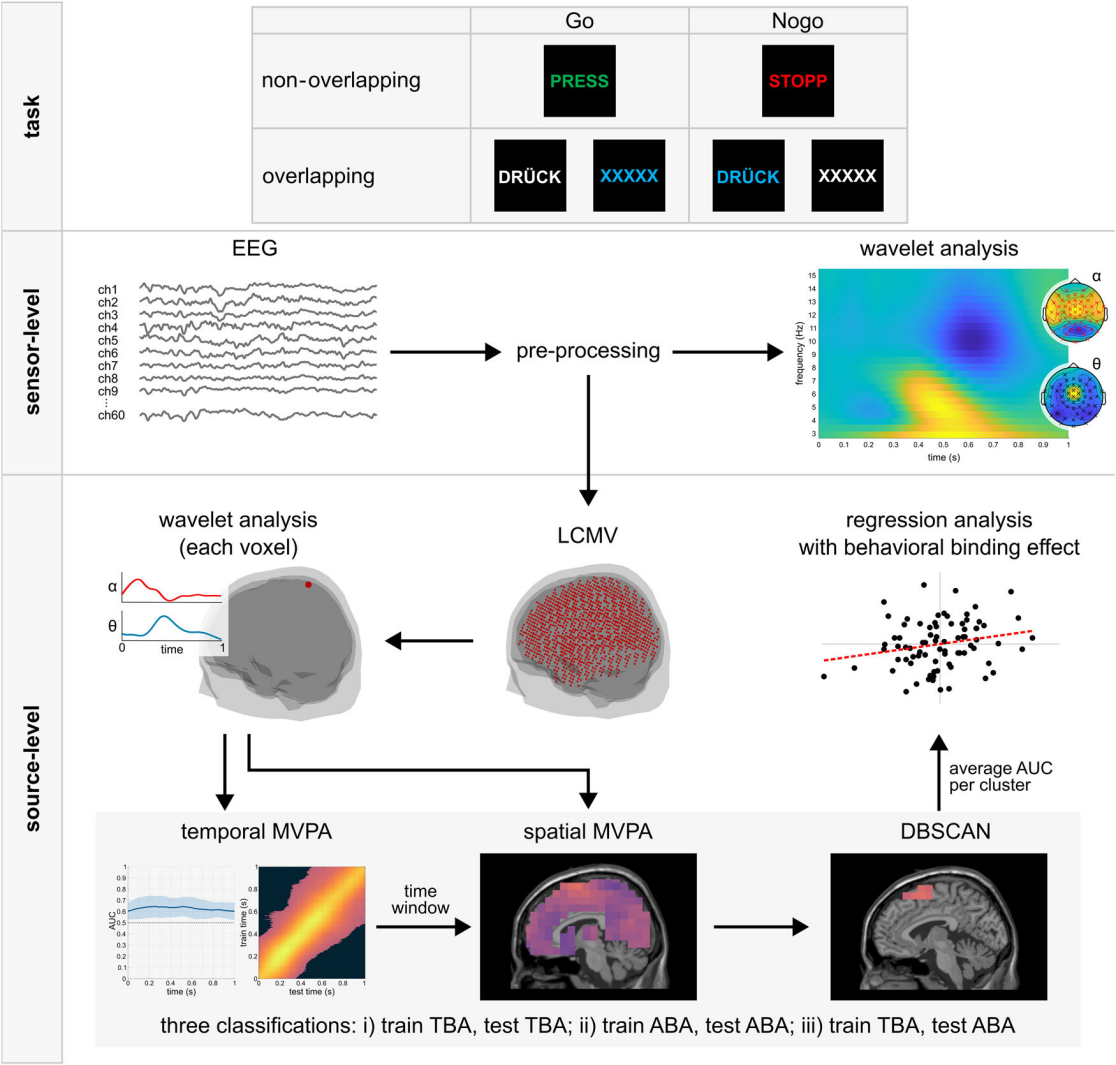
Visualization of the stimuli used in the task and the EEG analysis procedure. The top-panel shows the stimuli used in the non-overlapping and overlapping Go and Nogo conditions, respectively. The center panel shows the EEG analyses on the electrode-level. In the wavelet analysis, the difference between the overlapping and non-overlapping condition was assessed using cluster-based permutation testing. The lower panel visualizes the analyses on the source-level. The data were projected onto the source-level using LCMV beamforming. The head and brain images in this figure have been generated using the open-source FieldTrip software package, which was also used to run the analyses. Cerebellar and unlabeled voxels (AAL atlas) were excluded from this analysis. For each voxel, theta and alpha band activity time courses were computed with wavelet analyses. The temporal and spatial MVPA as well as the DBSCAN algorithm (gray box) were run for each of the three classifications. Subsequently, a regression analysis between the behavioral binding effect and the averaged AUC value in each cluster was conducted.

We investigate this hypothesis by combining EEG-beamforming methods and multivariate pattern analysis (MVPA) with a focus on theta and alpha band activity (TBA and ABA). TBA is relevant because the biophysical principles of low-frequency/high-amplitude oscillations are optimally suited to enable long-range integration processes^17,18^. In fact, they have already been shown to be involved in perception-action integration as conceptualized by TEC^9,13^. Yet, as regards the inhibition of responses, not only TBA^19^ but also ABA is relevant^20^. Both frequencies seem to be differentially engaged during response inhibition, depending on the necessity of reconfiguring stimulus-response associations while deciding whether or not to respond^12,13^. This suggests that it is not sufficient to examine the representational dynamics across different functional neuroanatomical structures within only one frequency band (i.e., TBA or ABA). Rather, representational dynamics must be examined across frequencies with the question of whether representations of stimulus-response bindings are “exchanged” between frequency bands to accomplish goal-directed behavior through the management of event files. To this end, participants completed a Go/Nogo task derived from the TEC framework. The stimuli consisted of different combinations of letters and font colors. In this way, two different conditions of Go or Nogo trials were created, which either had completely different features (letters, color) in Go and Nogo trials (non-overlapping condition) or shared features between Go and Nogo trials (overlapping condition; see Fig. 1). Thus, in the overlapping condition, reconfiguration of representations of the stimulus-response bindings was required, whereas in the non-overlapping condition such a reconfiguration was not required^14^. We used beamforming^21^ to reconstruct TBA and ABA time courses at their sources from the EEG data recorded during the Go/Nogo task. MVPA approaches were applied based on the source-reconstructed TBA and ABA time courses to analyze whether cortical regions concomitantly involved in response inhibition reveal similar dynamics of mental representations of stimulus-response bindings. The goal of the study was to reveal, which neuroanatomical regions within an activation pattern contribute strongest to the discrimination between different complexity levels of perception-action integration and whether a transfer of representations between TBA and ABA enables the dynamics driving goal-directed actions.

An overview of the neurophysiological analysis steps as a brief summary of the methods is provided in Fig. 1 (for details see the methods section at the end of the manuscript).

## Results

### Behavioral results

Shapiro-Wilk tests^22^ showed normal distribution of the Nogo false alarm rates in the overlapping condition (*p* = 0.343). However, none of the other behavioral outcome variables were normally distributed (*p* ≤ 0.038). Therefore, the comparisons between the conditions were performed using Wilcoxon tests. The Go hit rates were significantly higher in the non-overlapping (99.3 ± 0.9%) than in the overlapping condition (98.7 ± 1.4%; Z = −5.17, *p* < 0.001, *r* = −0.581). Furthermore, the Go reaction times were significantly faster in the non-overlapping (439 ± 52 ms) than in the overlapping condition (450 ± 54 ms; Z = −4.29, *p* < 0.001, *r* = −0.483). Importantly, the Nogo false alarm rate was significantly higher in the overlapping (37.5 ± 17.1%) than in the non-overlapping condition (2.2 ± 3.0%; Z = −7.72, *p* < 0.001, *r* = −0.869).

### Sensor-level activity

**Fig. 2:**
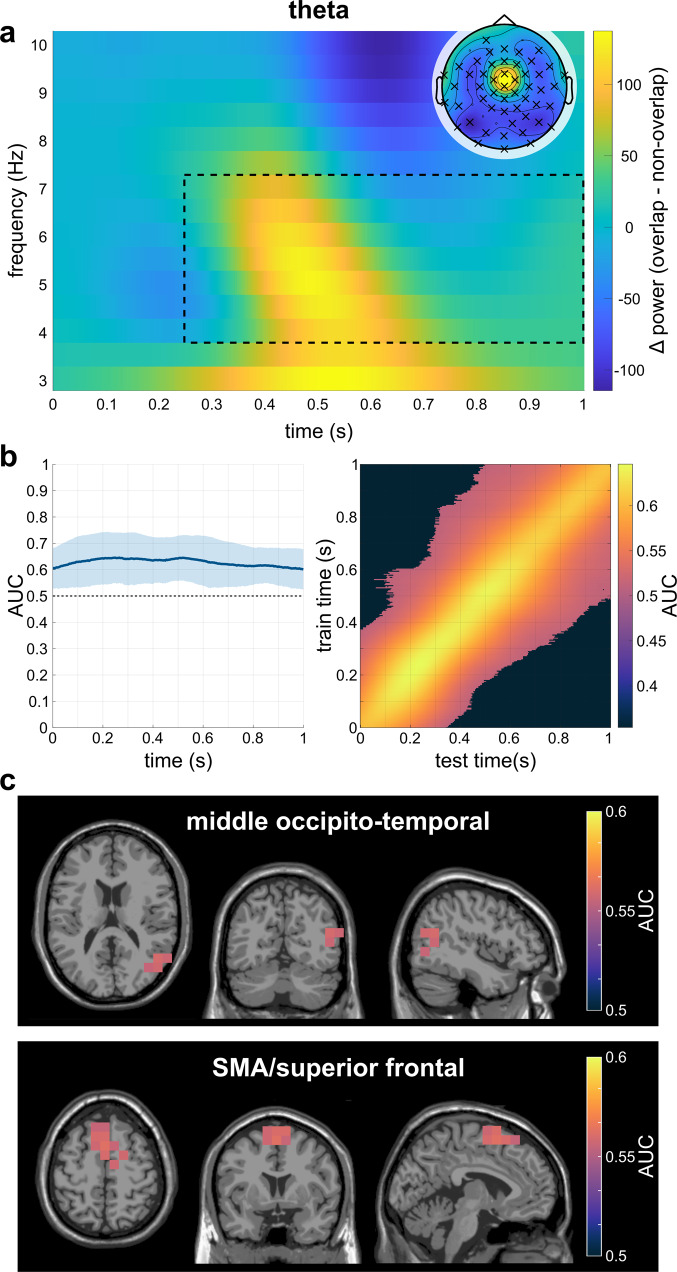
Results of the analyses on theta band activity of N = 79 subjects. **a** Time-frequency representation of the theta-band power difference on the significant electrodes on the sensor-level. The significant time frame is highlighted by the dotted black square, while the significant electrodes are highlighted in the topographic plot. **b** Results of the temporal MVPA. The plot on the left shows the AUC curve over time while the right plot shows the temporal generalization. The shaded area shows the standard deviation of the AUC. **c** Clusters of best performance in the top 2% of the AUC values of all significant voxels as identified by the DBSCAN algorithm.

**Fig. 3:**
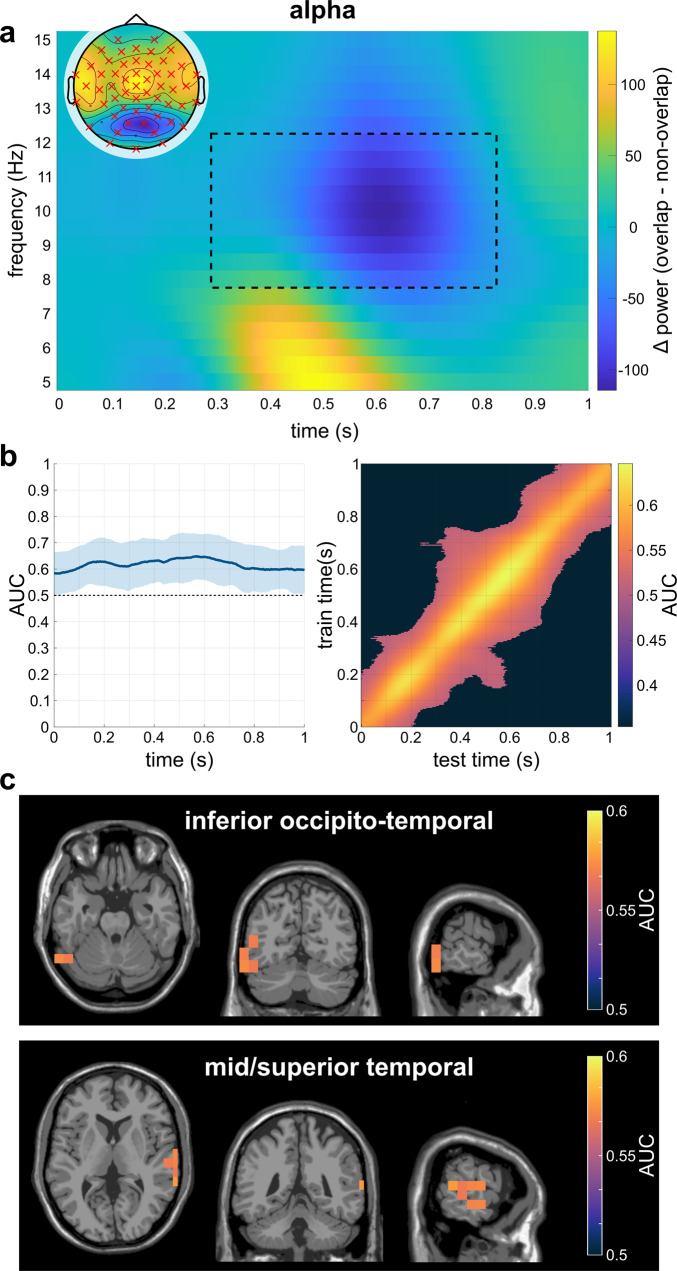
Results of the analyses on alpha band activity of N = 79 subjects. **a** Time-frequency representation of the alpha-band power difference on the significant electrodes on the sensor-level. The significant time frame is highlighted by the dotted black square, while the significant electrodes are highlighted in the topographic plot. **b** Results of the temporal MVPA. The plot on the left shows the AUC curve over time while the right plot shows the temporal generalization. The shaded area shows the standard deviation of the AUC. **c** Clusters of best performance in the top 2% of the AUC values of all significant voxels as identified by the DBSCAN algorithm.

A cluster-based permutation test on the theta frequency band (4–7 Hz) power between the overlapping and non-overlapping condition on the sensor-level revealed a significant positive difference (overlapping > non-overlapping); *t*_*sum*_ = 21489.55, *p*_*cluster*_ = 0.002. A cluster in the observed data extended from approximately 250 to 1000 ms (relative to stimulus onset) and extending across almost all EEG electrodes. The topographic distribution shows that the effect in the theta frequency band was most pronounced at central electrode locations (Fig. 2a). In the alpha-frequency band (8–12 Hz), cluster-based permutation testing on the sensor-level between the overlapping and non-overlapping condition revealed a significant negative difference (overlapping < non-overlapping); *t*_*sum*_ = −17856.50, *p*_*cluster*_ = 0.002. A cluster in the data was observed between 290 and 830 ms (relative to stimulus onset) and spanning across almost all EEG channels. The topographic map highlights that the largest difference in alpha-band activity between the two conditions was located at occipito-central electrodes (Fig. 3a).

### MVPA on source-level theta-band activity

The MVPA on the source-reconstructed theta power time course (i.e., averaged over the 4-7 Hz frequency bands) with theta-band training and prediction sets and subsequent cluster-based permutation testing showed that at all time points during the trial (0–1000 ms), the MVPA was able to distinguish between the overlapping and the non-overlapping condition significantly above chance level. The AUC values ranged from 0.601 to 0.646 (AUC_mean_ = 0.628; *p*_*cluster*_ < 0.001; Fig. 2b). The temporal generalization had an average duration of about 663 ms around the diagonal. 66% of the classifications yielded a significant AUC value, with AUC_mean_ = 0.57 for the significant classifications. Subsequently, the time points at which the conditions differed significantly (i.e., the entire trial) were submitted to a spatial MVPA as features, resulting in AUC values for all individual voxels. The AUC values for each voxel were tested against the chance level of 0.5 in a cluster-based permutation test, where all voxels exhibited a significantly positive performance difference (*t*_*sum*_ = 8232.07, *p*_*cluster*_ = .002; for voxels with a classification performance significantly above chance level: AUC_max_ = 0.563, AUC_min_ = 0.524, AUCmean = 0.541; Supplementary Fig. 1). The DBSCAN analysis on the AUC-values in the individual voxels revealed that within the top 2% of the AUC values, voxels within two clusters seemed to contribute most to the MVPA performance. One cluster was constituted by voxels in the right hemispheric middle temporal and occipital cortex (BA19), whereas the voxels in the other cluster were located in the left and right-hemispheric supplementary motor area (SMA) and the frontal superior cortex (BA6, BA8; Fig. 2c).

### MVPA on source-level alpha-band activity

The MVPA on the source-reconstructed alpha power time course (i.e., averaged over the 8–12 Hz frequency range) with alpha-band training and prediction sets and subsequent cluster-based permutation testing showed that at all time points during the trial (0–1000 ms), the MVPA was able to distinguish between the overlapping and the non-overlapping condition significantly above chance level (*p*_*cluster*_ < 0.001; AUC_max_ = 0.647, AUC_min_ = 0.582, AUC_mean_ = 0.616; Fig. 3b). The temporal generalization had an average duration of about 429 ms around the diagonal. 43% of the classifications yielded a significant AUC value, with AUC_mean_ = 0.56 for the significant classifications. Subsequently, the time points at which the MVPA differed significantly between both conditions (i.e., the entire trial) were submitted to a spatial MVPA as features in order to obtain an AUC value for each individual voxel. The resulting AUC values for each voxel were tested against the chance level (0.5) in a cluster-based permutation test, where all voxels exhibited a significantly positive performance difference (*t*_*sum*_ = 9767.12, *p*_*cluster*_ = 0.002; for voxels with a classification performance significantly above chance level: AUC_max_ = 0.577, AUC_min_ = 0.535, AUC_mean_ = 0.553; Supplementary Fig. 1).

The subsequent DBSCAN analysis on the AUC-values in the individual voxels revealed that the voxels with the highest performance in the MVPA were located in two clusters: one cluster was comprised of voxels in the left-hemispheric middle and inferior occipital and temporal cortex (BA19), the second cluster was located in the right-hemispheric middle and superior temporal cortex (BA21, BA22; Fig. 3c).

### MVPA with source-level TBA to ABA prediction

**Fig. 4:**
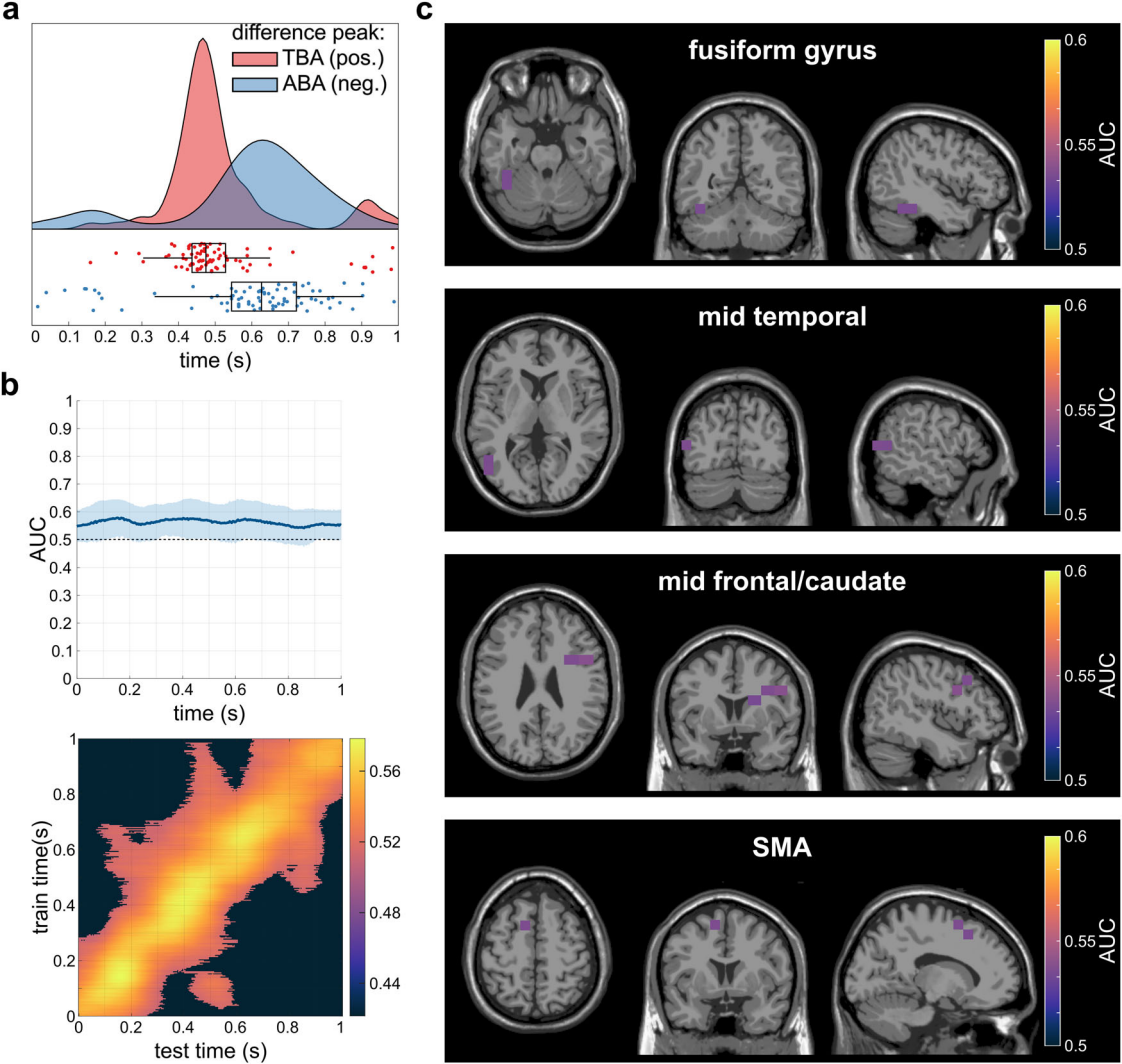
Results of the analyses on the relationship between theta and alpha band activity of N = 79 subjects. **a** Temporal distribution of the difference peaks in theta band activity (red) and alpha band activity (blue). Figure parts **b** and **b** show the results of the classification theta band activity to alpha band activity. The MVPA was run with a TBA training set and an ABA test set. **b** Results of the temporal MVPA. The upper plot shows the AUC curve over time (with the shaded area showing the standard deviation) while the lower plot shows the temporal generalization. **c** Clusters of best performance in the top 2% of the AUC values of all significant voxels as identified by the DBSCAN algorithm.

A MVPA with TBA training data and ABA testing data was conducted to reveal overlapping patterns between the different frequency bands. The TBA training was applied to the ABA data during testing, as the peak of power difference in TBA preceded the peak of power difference in ABA in the time-frequency decomposition on the sensor level (see Fig. 2a, b). In order to statistically evaluate the temporal difference of power peaks between TBA and ABA, the largest local maxima within the two frequency bands in the time period from 0 to 1 s were determined for each participant. Shapiro-Wilk testing showed that the data was not normally distributed (*p* < .001). A Wilcoxon signed-rank test showed that there was a significant difference between the temporal location of power peaks in TBA and ABA (*p* = 0.01, Z = −2.31), indicating that the TBA peak (*M* = 0.52 s, *SD* = 0.16 s) was significantly earlier than the ABA peak (*M* = 0.59 s, *SD* = 0.21 s; Fig. 4a). Individual data points on this parameter are given in Supplementary Data 1.

The MVPA on the source-reconstructed power time course with theta-band training and alpha-band prediction sets and subsequent cluster-based permutation testing showed that the MVPA was able to distinguish between the overlapping and the non-overlapping condition significantly above chance level over the entire trial duration (*p*_*cluster*_ < 0.001; AUC_max_ = 0.578, AUC_min_ = 0.541, AUC_mean_ = 0.562; Fig. 4b). The temporal generalization had an average duration of about 540 ms around the diagonal. Additionally, there was a significant off-diagonal AUC cluster within 0-200 ms training (TBA)/400–600 ms testing (ABA) time. 54% of the classifications yielded a significant AUC value, with AUC_mean_ = 0.54 for the significant classifications. Next, a spatial MVPA was performed using the significantly distinguishable time points (i.e., the entire trial) as input features. The resulting AUC values for each voxel were tested against the chance level of 0.5 in a cluster-based permutation test, where almost all voxels exhibited a significantly positive performance difference (*t*_*sum*_ = 5087.23, *p*_*cluster*_ = .002; for voxels with a classification performance significantly above chance level: AUC_max_ = 0.540, AUC_min_ = 0.510, AUC_mean_ = 0.523). A subsequent DBSCAN analysis on the AUC-values in the individual voxels revealed that the voxels with the highest performance in the MVPA were located in four clusters (Fig. 4c): one cluster was comprised of voxels in the left-hemispheric fusiform gyrus (BA37), the second cluster was located in the left-hemispheric middle temporal cortex (BA21), the third cluster was composed of right-hemispheric voxels in the middle frontal, caudate, inferior opercular frontal and the precentral cortex (BA46), and the fourth cluster was located in the left-hemispheric supplementary motor area (BA6).

### Relationship between behavioral and neurophysiological parameters

Starting with the AUC values of the obtained eight clusters (2 clusters in TBA, 2 clusters in ABA, 4 clusters in TBA-to-ABA) as predictors, the set of predictors could be reduced to two clusters (superior frontal/SMA theta cluster, middle frontal theta-alpha cluster) by the backward elimination procedure during the multiple regression analysis. The obtained regression model was statistically significant (adjusted R^2^ = 0.061, F(2,76) = 3.54, *p* = 0.034). The data contained no outliers (Std. Residual Min = −2.26, Std. Residual Max = 3.03) and met the assumption of independent errors (Durbin-Watson value = 1.87). Tests to determine whether the data met the assumption of collinearity showed that multicollinearity was not a concern in any of the steps of the backward elimination procedure (VIF ≤ 1.79). Only the mean AUC value in the middle frontal theta-alpha cluster significantly predicted the binding effect in false alarm rates in Nogo trials (β = 0.256, *p* = 0.025), whereas the mean AUC value in the superior frontal/SMA theta cluster did not ((β = −0.196, *p* = 0.084). In other words, the higher the classification accuracy of the transfer of representations from theta to alpha frequency band, the larger the behavioral binding effect in Nogo false alarm rates (i.e., the larger the difference in false alarm rate in overlapping vs. non-overlapping Nogo trials).

## Discussion

The current study aimed to identify the time periods and brain regions, in which differences in the mental representation of different perception-action integration demands in response inhibition are particularly pronounced and in which a potential “transfer” of these mental representations between frequency bands occurs. For this purpose, subjects performed a modified Go/Nogo task, where one condition only required retrieval of perception-action integration (non-overlapping condition), while the other condition required reconfiguration of already formed perception-action bindings (overlapping condition)^14^. Based on the source-reconstructed time-frequency data, differences in the spatial representation of the conditions over time in the alpha and theta frequency bands were examined with an MVPA approach. A further (spatial) MVPA served to investigate differences in the time course of the representation of the conditions over brain regions. These approaches were subsequently also adapted to investigate the interleaving of different frequency bands and thus the transfer of mental representations of the different event file coding requirements between them, which seems particularly relevant given the theoretical assumptions of TEC^2,3^ and previous findings^12,13^.

Concerning the behavioral data, we replicate previous studies^11–15^, i.e., participants’ performance was better in the non-overlapping condition compared to the overlapping condition. This was particularly evident in the Nogo false alarm rates, which were considerably higher in the overlapping condition than in the non-overlapping condition. These results are consistent with the theoretical assumptions of the TEC predicting that feature overlap between Go and Nogo trials, i.e., using the same stimulus features for different response requirements, leads to partial repetition costs that are reflected in performance impairments^2,5^. Also for the sensor-level neurophysiological data, the comparison of the Nogo conditions replicated the pattern of results in the time-frequency data of previous studies^12,13^, i.e., higher TBA in the overlapping compared to the non-overlapping condition, thus the TBA binding effect reflecting more demanding event file (re)configuration processes. This finding is well in line with the general notion of stronger TBA during cognitive control demands^9,18,19^. The ABA binding effect occurs at its maximum after the TBA binding effect maximum and has an opposite pattern compared with the TBA binding effect. Previous findings suggest that ABA likely reflects inhibitory gating mechanisms^20,23,24^ that are more pronounced in the non-overlapping condition compared with the overlapping condition. This implies that task-irrelevant processes are more strongly inhibited in the non-overlapping condition, which facilitates the processing of relevant information such as the required event file, probably due to the unambiguous stimulus categorization^23,24^. On the other hand, the less pronounced inhibitory gating in the Nogo condition with feature overlap with Go trials might reflect a broader encoding of information^25,26^ which includes potentially irrelevant event files.

### MVPA classification within frequency bands

The MVPA on the source-level data, i.e., the power time courses in each defined voxel, yielded successful classifications across time within the alpha and theta frequency bands, respectively. In both MVPAs, the representations of the non-overlapping and overlapping event files could be distinguished over the entire time window. Moreover, there was also a successful temporal generalization MVPA in both frequency bands, indicating that the representational differences in event files between the Nogo conditions were sustained for several hundred milliseconds within both frequency bands^27^. Importantly, for the theta frequency band, the spatial MVPA yielded the best classification results in the right occipito-temporal cortex as well as in the supplementary motor area (SMA) and superior frontal areas. Particularly in the SMA, differences in TBA due to event file coding demands and inhibitory performance have been localized in previous studies, but thus far only with respect to the power of activity (differences)^12,13,28–30^ and therefore unrelated to effects related to representational content. Based on these previous results on the relevance of the SMA for event file coding and response inhibition, the current results demonstrate that event file binding representational dynamics reflected in the theta frequency band significantly differ in their time course depending on the complexity of perception-action integration. Intriguingly, occipito-temporal TBA power differences were also associated with event file coding dynamics in response inhibition in a previous study, however, in the pre-trial interval and not after stimulus presentation^13^. Occipito-temporal TBA is related, for example, to color categorization in the ventral stream^31–33^, which in the present paradigm is of crucial importance for the classification of the stimulus as Go or Nogo due to overlapping stimulus features (e.g., color). Moreover, attentional selection processes have been attributed to this region^34–37^. It is thus reasonable that the dissimilarity of the mental representation of the different Nogo conditions is particularly evident in this area, which encodes a crucial feature for distinguishing the conditions. Of note, event file coding has been shown to be dynamic in a sense that brain structures specialized for stimulus features are involved in event file coding processes^7^. The current findings provide an important extension to this knowledge by showing that these dynamics are supported by representational content coded in TBA.

Regarding the alpha frequency band, the spatial MVPA revealed the best classification results in the left occipito-temporal cortex and in the right superior and middle temporal gyri. Among other things, these areas are associated with encoding and retrieval of short-term memories^38–40^, as which event files can also be understood due to their episodic nature^3^. These areas are also part of the ventral stream encoding stimulus information such as color^31–33^ and thus a decisive perceptual feature to inform response execution or inhibition in the current study. One characteristic of ABA is that it represents an inhibitory filter, in the sense that ABA reflects inhibition of irrelevant information and processes (i.e., top-down control)^20,23,24^. The current findings on the most successful classification of the time courses of ABA into either a representation of no overlap or overlap in areas responsible for retrieval of episodic memories (i.e., event files) and color categorization (which is a decisive feature in the current task) suggest that particularly top-down control over these two cognitive functions is adjusted depending on the degree of overlap. Based on previous findings^12,13^ and the current results of the sensor-level data on the direction of power differences, it can be assumed that the control of these functions in terms of suppression is more pronounced (as reflected in higher ABA) in situations requiring pure retrieval compared to situations requiring reconfiguration. Thus, it is likely that in these situations with pure retrieval, the recall of irrelevant episodic memory content is suppressed, i.e., retrieval of irrelevant event files is prevented.

### MVPA classification between frequency bands

Based on the timing of the power difference peaks in previous studies^12,13^, which was replicated and statistically validated by the current results on the sensor level, the MVPA for linking TBA and ABA was performed with TBA as training data and ABA as test data. Notably, the differential voxel patterns that distinguished between conditions in TBA were also relevant to distinguish between conditions in ABA over the entire time course. Given the timing of the observed power difference peaks, this not only indicates similarities in both frequency bands regarding the mental representations involved in the binding effect, but suggest a transfer of mental representations from TBA to ABA during the whole time period of a trial. Furthermore, the temporal generalization of MVPA from TBA to ABA revealed that representational differences from TBA could also be found in ABA over a longer time course^27^. Moreover, TBA right after the stimulus presentation could predict ABA around the time of the maximum ABA binding effect, indicating a recurrent activation of mental representations^27^. This finding further supports the presumed direction of transfer from TBA to ABA. The data exhibited an interleaved patterning of representations in TBA and ABA in several brain regions, some of which overlapped with brain regions, in which representations of varying degrees of overlap also differed within each frequency band. Importantly, the relevance of the SMA to event file coding was evident again, as was the case when the theta frequency band was considered separately. Additionally, these results provide converging evidence across methods^12,13,28^, demonstrating that the involvement of the SMA in event file coding can be considered a robust finding. The involvement of the left posterior middle temporal gyrus as well as the left fusiform gyrus also fit into the previously drawn picture, as these two areas map parts of the ventral stream associated with color categorization and semantic categorization (e.g., of written letters), respectively^41–43^. Both colors and letters are features with a central importance for the assignment of the presented stimuli to an action alternative (reaction vs. non-reaction). However, the largest contiguous cluster of voxels with a large extent of transfer of mental representations between TBA and ABA was found in the right middle frontal gyrus (rMFG), which is, among others, associated with episodic memory retrieval^44^ and its monitoring^45^. In particular, the monitoring of memory retrieval likely differs substantially between conditions, as the requirements for this monitoring are probably higher in conditions requiring reconfiguration than in conditions where retrieval of the initially activated event file leads to success. The interplay of TBA and ABA in this process underpins its particular relevance to successful inhibition under varying event file coding demands. Importantly, only the extent of the transfer of mental representations from TBA to ABA in the rMFG predicted behavioral performance, that is, a more successful transfer of mental representations from TBA to ABA was associated with an increase in the behavioral binding effect (i.e., an increase in false alarm rate). Thus, it could be questioned whether the close intertwining of the two frequency bands is beneficial when it comes to event file coding. It is particularly plausible that there is a transfer of mental representation from TBA to ABA against the theoretical background of TEC and the closely related BRAC framework^4^. After stimulus presentation, automatic activation of event file content is triggered^3,46^, which might be reflected in the theta frequency band. Thus, TBA might be of functional relevance for the binding and retrieval of event files, probably due to its biophysical principles providing communication across large spatial distances^17,47^. However, in order to then make a selection between competing event files and choose the one that is appropriate given the current requirements, top-down control is needed to dynamically handle event files, which might be represented in the alpha frequency band. Thus, ABA may modulate processes of event file coding, retrieval and reconfiguration, which are most likely reflected by TBA. This may potentially even explain the ambiguously beneficial effects of this interplay on behavior, as the closer interleaving between the two frequency bands may suggest that the top-down control reflected in ABA “takes over” the mental representations of TBA before the reconfiguration of the event files is adequately completed, which then increases the binding effect, i.e., the magnitude of the effect of the degree of feature overlap. The pattern of findings corroborates recent theoretical work on the role of ABA and TBA during action control according to which it is the interplay of both frequency bands that is central for the dynamic management of integrated perception-action representations^48^. Future studies, for example using methods to examine effective connectivity, may investigate the precise transfer of information between brain regions involved. Against the background of the theoretical framework^1,3,4^, a further question would be to what extent different features differ in their contribution to costs due to feature overlap. Although, according to the theoretical background^1,3,4^, all present features are bound in the event file, a weighting of features is assumed. Thus, future studies may examine in how far there are specific combinations/contributions of low-level features (e.g., color, visual appearance, word vs. non-word, meaning) possibly determining representational dynamics more than other low-level features – for example using representation similarity analysis (RSA)^49,50^.

## Conclusions

Overall, the prediction of mental representations encoded in the alpha frequency band from mental representations encoded in the theta frequency band reveals a close interplay of both frequency bands in perception-action binding and retrieval in response inhibition. Conceptually, this interplay implies different functions of both frequency bands in the context of event file coding: while the theta frequency band seems to represent general event-file associated processes such as binding, retrieval, and reconfiguration, the alpha frequency band seems to represent a top-down control over these processes, implying that the alpha frequency band may play an important role in the *handling* of perception-action representations These functions can be interpreted against the theoretical background of the BRAC framework, which considers top-down processes to have an important role in the dynamic management of perception-action representation during their establishment (binding) and their retrieval.

## Materials and methods

### Sample

We assessed *N* = 93 healthy participants aged 20 to 30 years between April 2019 and June 2021. The task was conducted as part of a larger data collection, consisting of two appointments of 3.5 hours each. Participants were recruited as a convenience sample via advertisements and an in-house database. After exclusions due to not meeting inclusion criteria (*N* = 11), technical problems (*N* = 1), and outlier correction of the behavioral data (*N* = 2), the final sample consisted of 79 subjects (33 females; age: 24.0 ± 2.8 years; IQ: 111 ± 13). There is a partial overlap of this sample with previous studies of our workgroup investigating the same task^11–13,15,51^. All included participants reported the absence of psychiatric or neurological disorders during a brief telephone screening, which was underpinned by the *Adult Self-Report* (ASR/18-59)^52^ and the *Alcohol, Smoking and Substance Involvement Screening Test* (ASSIST)^53^. Before study procedures started, the subjects provided written informed consent. They received financial compensation of 60 EUR or course credits after completing both appointments of the larger data collection. The local ethics committee of TU Dresden approved the study.

### Task

To assess perception-action integration during response inhibition, participants were instructed to perform a TEC Go/Nogo task^14^. The task consisted of Go and Nogo trials, which contained either overlapping features or non-overlapping features. The conditions with overlapping features were characterized by the fact that their properties (color or letters) were used for both the Go and the Nogo conditions. Since the Go condition required a response, whereas Nogo trials required to inhibit the response, this overlap in stimulus features led to partially overlapping event files (i.e., coupling between stimulus features and responses), which should induce a higher false alarm rate during Nogo trials. The conditions with non-overlapping features, on the other hand, contained features that were exclusively used for either Go or Nogo trials. In detail, non-overlapping Go trials were defined by the green-lettered word “PRESS”, whereas non-overlapping Nogo trials consisted of the red-lettered word “STOPP”. There were two types of overlapping Go and Nogo trials each. In the first overlapping Go condition, the word “DRÜCK” (German word for “press”) was shown in white letters. The second overlapping Go condition consisted of the letters “XXXXX” presented in blue. Regarding the Nogo condition, the overlapping trials displayed either the word “DRÜCK” presented in blue font or the letters “XXXXX” shown in white font. Participants were asked to respond to Go trials by pressing the space key with their right index finger as fast as possible. Prior to the task, a training session was conducted to familiarize each participant with the task. In the actual task, 196 stimuli were shown for each Go condition (overlapping and non-overlapping), whereas 84 trials were presented for each Nogo condition (overlapping and non-overlapping). This 70:30 ratio of Go vs. Nogo stimuli was chosen to create a prepotent response tendency^54^. The task consisted of seven blocks of equal length. In all blocks, every condition was presented with equal frequency and in a pseudorandomized order. Participants could decide on the length of the break in between the blocks. Every stimulus was presented for a duration of 450 ms. A trial hence started with stimulus onset and ended either with the participants’ response or after 1700 ms if no response occurred. The inter-trial interval was jittered between 700 and 1100 ms. When no stimulus was shown, a white fixation cross was presented in the center of the screen.

For further analyses, the two types of overlapping Go conditions were combined. The same was done for the Nogo conditions. For the behavioral analysis, the false alarm rate (given as percentage) was averaged for overlapping and non-overlapping Nogo trials each. Furthermore, the reaction time (RT; given in ms) was averaged for overlapping and non-overlapping Go trials, respectively. For the neurophysiological analysis, only Nogo trials were considered, because we were specifically interested in event file dynamics during inhibitory control.

### EEG recording and analysis

While the participants were performing the TEC Go/Nogo task, their EEG was recorded with 60 equidistant Ag/AgCl electrodes, which were embedded in an elastic cap (EasyCap Inc.). The reference electrode was located at θ = 90, ϕ = 90, the ground electrode at θ = 58, ϕ = 78. For the recording, the software BrainVision Recorder 2.1 (Brain Products) was used. The EEG was measured with a sampling rate of 500 Hz and electrode impedances were kept below 5 kΩ. After recording, the data were pre-processed offline with the software BrainVision Analyzer 2.1 (Brain Products). We reduced the sampling rate from 500 Hz to 256 Hz and applied an infinite impulse response (IIR) filter from 0.5 to 40 Hz and a notch filter of 50 Hz. Data were then referenced to the average of all electrodes. With a first raw data inspection, we removed technical and muscular artifacts. A subsequent independent component analysis (ICA; restricted infomax method) served to detect and exclude recurring artifacts, such as blinks and pulse artifacts. Afterwards, we segmented the data locked to the onset of the overlapping and non-overlapping Go and Nogo trials, respectively. Only correctly rejected Nogo trials with no response until 1500 ms after stimulus onset were included in the further data processing and analysis. The segments had a size of 4000 ms and encompassed the time interval from 2000 ms before to 2000 ms after stimulus onset. Remaining artifacts were removed by an automated artifact rejection that detected segments containing amplitudes below -200 μV or above 200 μV and segments with activity below 0.5 μV in an interval of 100 ms. To complete EEG data preparation, a baseline correction was performed in the time window from −200 ms to stimulus onset.

### Time-frequency analysis

We conducted a time-frequency decomposition on each participant’s single-trial electrode-level EEG data and averaged it across all trials based on the condition. Accordingly, time-frequency activity was computed for correctly rejected overlapping and non-overlapping Nogo trials. We applied a Morlet parameter of 5. Subsequently, we compared the participants’ average theta band power (4–7 Hz) in the time window from 0 to 1000 ms after stimulus onset between overlapping and non-overlapping Nogo trials with a cluster-based permutation test^55^. First, paired *t*-tests were calculated to compare both conditions at each electrode. If at least two pairs of neighboring electrodes showed a significant *t*-value (*p* < 0.05), they were considered part of a sample cluster. The sum of the *t*-values in each cluster represented the cluster-level statistics. As a second step, the significance probability was calculated with the Monte Carlo method. From both conditions combined, 1000 random draws of trials were tested for significant differences to approximate the reference distribution. Calculating the portion of randomly drawn trials that showed a larger test statistic than the observed trials resulted in the significance probability, i.e., the *p*-value. If a cluster reached a *p*-value below 0.05, it was considered to significantly differ in activity between conditions. By this means, channels with significant differences in theta activity between the overlapping and non-overlapping Nogo trials could be identified. This procedure was repeated for the participants’ average alpha band power (8–12 Hz). The purpose of the procedure was to check whether there were any differences at all in the theta and alpha band between the conditions and clusters. This was done to ascertain, whether the subsequent steps were justified, especially because the MVPA applied later is suggested to complement, not replace, classical statistical analyses^56^.

### Beamforming analysis

Since we were interested in the source-reconstructed time-frequency data of the participants, we applied a Linear Constraint Minimum Variance (LCMV) beamformer^21^ on the pre-processed single-trial EEG data. LCMV beamforming allows reconstruction of the time series data in the source regions from the recorded scalp activity. It works by multiplying a spatial filter (computed based on the covariance matrix of the time-locked averaged data of each condition and cluster) with the EEG data. The LCMV beamformer was computed for each subject and each condition (overlapping and non-overlapping Nogo trials) from the information of all electrodes in the time frame from -2 to 2 s relative to stimulus onset. For the LCMV computation, a leadfield of 1 cm grid resolution was first created based on the Montreal Neurological Institute (MNI) coordinate system provided in the FieldTrip toolbox. For each grid point (i.e., voxel) of the brain labeled in the Automatic Anatomical Labeling atlas (AAL)^57^, the time series data were reconstructed using the LCMV beamforming. In addition to unlabeled voxels, voxels located in cerebellar structures were excluded from the source analysis, resulting in time courses for a total of 1254 voxels. In order to receive TBA and ABA activity time courses for each source, we computed a time-frequency analysis in a frequency range from 2 to 15 Hz in steps of 0.5 on the time series data of each voxel. We used a Morlet parameter of 5. For theta power, activity from 4 to 7 Hz was averaged; for alpha power, activity from 8 to 12 Hz was averaged. To reduce the data load, we restricted further analyses to the time window from stimulus onset until 1000 ms after stimulus onset. In conclusion, by applying the LCMV beamforming method we obtained the source-reconstructed theta and alpha power in each brain voxel per participant and trial at every time point from 0 to 1000 ms after stimulus onset.

### Multivariate pattern analysis (MVPA)

The goal of the study was to investigate the mental representations underlying stimulus-response binding via MVPA. In particular, we aimed at examining which concomitantly active brain regions (within a pattern of brain activity) contribute most to the classification into an overlapping vs. non-overlapping trial based on the source-reconstructed time-frequency information (i.e., the results of the LCMV beamforming). Furthermore, the transfer of representational processes between TBA and ABA was analyzed. To this end, three different classifications were investigated: (1) A classifier trained and tested on the source-reconstructed time course of TBA; (2) A classifier trained and tested on the source-reconstructed time course of ABA; (3) A classifier trained on the source-reconstructed time course of TBA and tested on the source-reconstructed time course of ABA. The order of the third approach (i.e., train on TBA and test on ABA) was chosen since previous analyses have shown that the theta power difference between the overlapping and the non-overlapping condition peaks before the alpha power difference after the presentation of Nogo stimuli in the TEC Go/Nogo task^13^. Thus, it would not be meaningful to train a classifier based on alpha band data and test it on theta band data.

For each of the three investigated approaches, we computed multivariate pattern analyses (MVPA) using the MVPA-light toolbox^56^ and trained a binary classifier to discriminate between the overlapping and non-overlapping condition. The chosen parameters and settings as well as the analysis steps are described in the following: In general, we adopted the default settings of the toolbox except for choosing a two-class L1-Support Vector Machine (SVM) classifier. We decided to apply the SVM classifier because it is more robust to outliers and therefore better suited to noisy and non-Gaussian data than the LDA classifier^56^. Furthermore, we used a cross-validation method with five folds. The MVPA analyses were performed on each participants’ dataset with the source-reconstructed time course in each of the 1254 voxels (derived from the LCMV processing step) as feature vector (i.e., as input). First, we performed an MVPA to investigate at which time points after stimulus presentation the classifier can discriminate between both conditions based on the pattern of the source-reconstructed time-frequency data (i.e., theta power and alpha power, respectively) across all voxels. The results were displayed via the area under the ROC curve (AUC). Additionally, a temporal generalization analysis was carried out with the same feature vector to obtain information on the temporal dynamics of the activation patterns. We performed cluster-based permutation tests (based on Wilcoxon tests with a threshold set at *p* = 0.05) to test at which time points the AUC differed significantly from the chance level (i.e., 0.5). To approximate the reference distribution, 1000 random draws were used. The statistical values of the cluster-based permutation tests were computed as the sum of all Wilcoxon-values within the time points.

For the significant time windows of the first MVPA, i.e., the time windows in which the AUC differed significantly from chance level, a spatial MVPA was performed. In the spatial MVPA, the classifier was trained with time (i.e., the time-frequency course) as feature vector. As the output, we received an AUC value for each voxel. The spatial MVPA thus analyzed the classification performance of each voxel based on the information about the source-reconstructed time-frequency course. To test in which voxels the AUC value differed significantly from chance level (i.e., a chance-level brain), we performed cluster-based permutation tests with the same parameters as described above. Importantly, however, from this analysis step onwards, we had to re-include previously excluded voxels (see section “Beamforming Analysis”). As the source-locations were flattened as one dimension of the MVPA analysis, a 3-dimensional structure had to be re-established for subsequent statistical comparisons. Thus, the AUC values for each voxel were re-inserted into the source data, and voxels at previously excluded positions were set to a value of 0, resulting in a total of 2020 voxels. The corresponding voxels in the chance-level brain were also set to a value of 0. As a final step, we investigated whether the voxels with the highest classification performance (i.e., the highest AUC values) form clusters and can thus be assigned to neuroanatomical regions. To this end, we applied a Density-Based Spatial Clustering of Applications with Noise (DBSCAN) algorithm^58^. In detail, the DBSCAN identified the top 2% of AUC values with at least one neighbor within a neighborhood search radius of ε = 1.5 * grid size that were located in gray matter regions labeled in the Automatic Anatomical Labeling atlas (AAL)^57^. The applied analysis steps thus served to reveal which neuroanatomical regions within an activation pattern contribute strongest to the discrimination between overlapping vs. non-overlapping conditions based on source-reconstructed time-frequency information.

### Statistics and reproducibility

For the behavioral data, the analyses of the Go and Nogo trials were performed separately. The Go conditions “white PRESS” and “blue XXXXX” were combined into one overlapping Go condition, while the Nogo conditions “blue PRESS” and “white XXXXX” were combined into one overlapping Nogo condition. Thus, the contrast between overlapping and non-overlapping conditions could be analyzed for the following parameters: accuracy of response (hit rate) and reaction times (RT) on Go trials, and false alarm rate on Nogo trials. The overlapping and non-overlapping conditions were compared using non-parametric Wilcoxon tests, as the Shapiro-Wilks tests^22^ were significant (see behavioral results). For significant comparisons, the effect size r was calculated according to Rosenthal^59^. For the neurophysiological analyses, only the Nogo trials were used because this condition measures response inhibition. For both the behavioral and neurophysiological data, the difference of overlapping minus non-overlapping condition is referred to as the binding effect. Moreover, we aimed to predict behavioral performance from the results of our spatial MVPA. Following the study of Petruo et al.^60^, multiple linear regression with a backward elimination procedure was used to find the optimal predictor set out of the clusters obtained in the spatial MVPA for predicting the binding effect in the false alarm rate of the Nogo trials. The mean AUC values of the clusters were used as predictors in the model. At each step, predictors were removed based on the change of the coefficient of determination R², until the elimination of another predictor would substantially decrease R^2^ (*p* < 0.10; default setting in SPSS).

The sample size is larger compared to previous studies using MVPA on data^11,60^ and comparably large compared to a recent study using MVPA in the same experimental paradigm^15^. Details regarding the statistics for the MVPA are given in the respective part of the methods section (see above). All data is available in OSF.

### Reporting summary

Further information on research design is available in the Nature Portfolio Reporting Summary linked to this article.

## Supplementary information

Supplemental Material

Description of Additional Supplementary Files

Supplementary Data 1

reporting summary

## Data Availability

Data can be downloaded from: 10.17605/OSF.IO/2HNYK.

## Code availability

Further custom code used to process the data can be found here: 10.17605/OSF.IO/2HNYK.
