# Supplementary material for: Interplay between alpha and theta band activity enables management of perception-action representations for goal-directed behavior
Source: Commun Biol. 2023 May 6;6:494. doi: 10.1038/s42003-023-04878-z (PMC10164171; doi:10.1038/s42003-023-04878-z)
Supplement: Supplementary file 1 — Supplemental Material [file 42003_2023_4878_MOESM1_ESM.pdf]

Supplementary Information

Interplay between alpha and theta band activity enables management of perception-action representations for goal-directed behavior

Paul Wendiggensen, Astrid Prochnow, Charlotte Pscherer, Alexander Münchau, Christian Frings, Christian Beste

Supplementary Figure 1. Visualization of the classification performance (AUC) in each voxel for the three MVPA analyses of N=79 subjects.

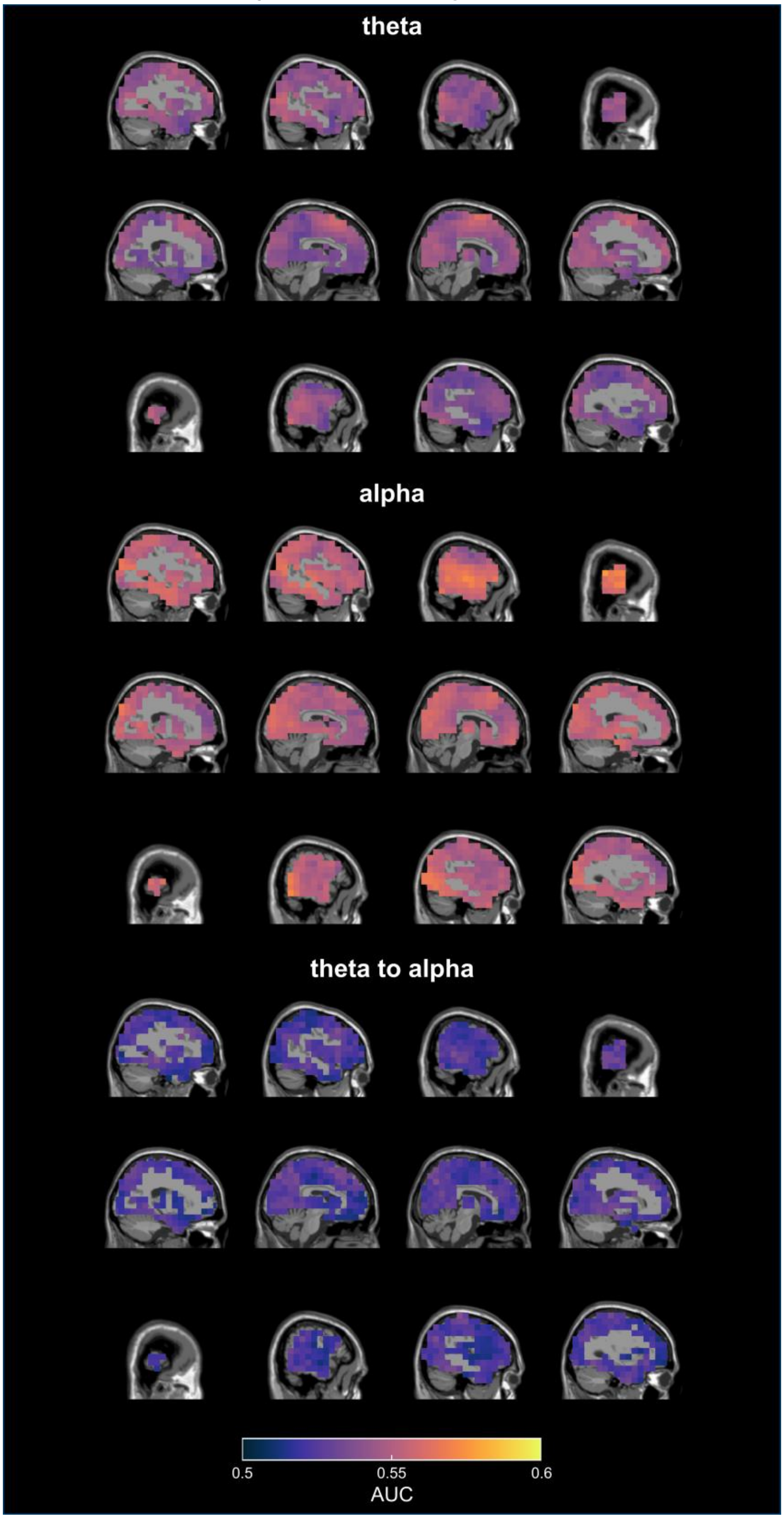

AUC values were thresholded to those voxels that showed significant performance above chance level in the respective cluster-based permutation tests. Each of the individual classifications is displayed by twelve (3x4) sagittal slices. The top plots show the classification performance in the MVPA with a TBA test and TBA training set, while the second set of plots shows the classification performance in the MVPA with an ABA test and ABA training set. The bottom plots show the classification performance in each significant voxel for the MVPA utilizing a TBA test and an ABA training set.
