# Supplementary material for: Interplay between alpha and theta band activity enables management of perception-action representations for goal-directed behavior
Source: Commun Biol. 2023 May 6;6:494. doi: 10.1038/s42003-023-04878-z (PMC10164171; doi:10.1038/s42003-023-04878-z)
Supplement: Supplementary file 2 — Description of Additional Supplementary Files [file 42003_2023_4878_MOESM2_ESM.pdf]

Description of Additional Supplementary Files

File name: Supplementary Data 1

Description: The source data behind the graph in Figure 4a.
